# 

**Published:** 1882-04

**Authors:** 


### EDITORIAL.

### ILLINOIS DENTAL SOCIETY.

The Eighteenth Annual Meeting of "The Illinois State Dental Society" will be held at Quincy on the second Tuesday (9th) of May, and will continue four days.

The dentists of Illinois and the neighboring States are cordially invited to be present. EDMUND NOYES, Sec'y.

### KANSAS STATE DENTAL ASSOCIATION.

LAWRENCE, Kansas, March 1, 1882.

DEAR DOCTOR:---The Eleventh Annual Meeting of the Kansas State Dental Association will be held in Topeka, Kansas, commencing Tuesday, May 2, 1882.

Members of the profession throughout Kansas and adjoining States, are urgently requested to attend this meeting, and to prepare essays and reports of cases in practice to be read at that time. Kansas dentists should place the meeting of the State Association first in importance, and, while giving due attention to local society meetings, ought to consider that the State organization requires their best aid and attendance.

Each member of the Association especially is expected to contribute to the success of the meeting by preparing himself upon some subject connected with the theory and practice of dentistry; and in order that the programme of the meeting may be arranged and distributed, it is requested that subjects of essays, etc., be sent to the Secretary at an early day.

The usual reduction in railroad fares and hotel rates will be secured, and there will be exhibited by dental dealers complete stocks of dental goods.

J. D. PATTERSON, Secretary, Lawrence, Kan.
A. H. THOMPSON, President, Topeka, Kan.

### THE DENTAL REGISTER,

A MONTHLY MAGAZINE DEVOTED TO THE INTERESTS OF THE

**DENTAL PROFESSION.**

*Terms Reduced to $2.00 Per Year. Single Copies, 25 Cents.*

**EDITED BY PROF. J. TAFT, D.D.S.**

—AND—

***Published by SPENCER & CROCKER, Ohio Dental Depot.***

The volume commences in January and closes in December.

The REGISTER being issued monthly is always fresh, therefore Indispensable to the practitioner who desires to keep posted up with the new inventions and improvements which are daily developed. Neither expense nor effort will be spared to give value and variety to its contents. Articles will be illustrated with well executed engravings whenever they will add to the interest or assist to explanation.

Its extensive circulation and frequency of issue make it one of the BEST DENTAL ADVERTISING MEDIUMS in the United States.

All subscriptions must begin with January or July.

Local or special notices, five lines or less, will be inserted for $3 each insertion; over five lines, 50 cts. per line.

All advertising bills payable in advance, or at furthest, after the issue of the first number containing the advertisement. All transient advertisements to be paid for in advance.

☞CONTRIBUTIONS SOLICITED.

Exclusively Editorial Communications should be addressed to the Editor.

The latest number of the REGISTER may be ordered with or without other goods at any time, and all letters should be addressed to

**SPENCER & CROCKER, Ohio Dental Depot, Cincinnati, Ohio.**

